# Serosurvey of *Treponema pallidum* infection among children with skin ulcers in the Tarangire-Manyara ecosystem, northern Tanzania

**DOI:** 10.1186/s12879-020-05105-4

**Published:** 2020-06-03

**Authors:** Clara K. C. Lubinza, Simone Lueert, Luisa K. Hallmaier-Wacker, Esther Ngadaya, Idrissa S. Chuma, Rudovick R. Kazwala, Sayoki G. M. Mfinanga, Klaus Failing, Christian Roos, Sascha Knauf

**Affiliations:** 1 National Institute for Medical Research, Muhimbili Medical Research Centre, P.O. Box 3436, Dar es Salaam, Tanzania; 2 College of Veterinary Medicine and Biomedical Sciences, Sokoine University of Agriculture, P.O. Box 3021, Morogoro, Tanzania; 3 Work Group Neglected Tropical Diseases, Infection Biology Unit, Deutsches Primatezentrum GmbH, Leibniz Institute for Primate Research, Kellnerweg 4, 37077 Goettingen, Germany; 4 Primate Genetics Laboratory, Deutsches Primatenzentrum GmbH, Leibniz Institute for Primate Research, Kellnerweg 4, 37077 Goettingen, Germany; 5 Muhimbili University of Health and Allied Sciences, P.O. Box 65001, Dar es Salaam, Tanzania; 6 School of Life Sciences and Bioengineering, Nelson Mandela African Institute of Science and Technology, Arusha, Tanzania; 7 Unit for Biomathematics and Data Processing, Faculty of Veterinary Medicine, Justus Liebig-University-Giessen, Giessen, Germany; 8 Gene Bank of Primates, Deutsches Primatenzentrum GmbH, Leibniz Institute for Primate Research, Kellnerweg 4, 37077 Goettingen, Germany; 9 Division Microbiology and Animal Hygiene, Department for Animal Sciences, Georg-August-University, Burkhardtweg 2, 37077 Goettingen, Germany

**Keywords:** *Treponema pallidum* subsp. *pertenue*, Yaws, Tropics, Africa, Nonhuman primates, Serology, Syphilis, Treponematoses

## Abstract

**Background:**

The first yaws eradication campaign reduced the prevalence of yaws by 95%. In recent years, however, yaws has reemerged and is currently subject to a second, ongoing eradication campaign. Yet, the epidemiological status of Tanzania and 75 other countries with a known history of human yaws is currently unknown. Contrary to the situation in humans in Tanzania, recent infection of nonhuman primates (NHPs) with the yaws bacterium *Treponema pallidum* subsp. *pertenue* (*TPE*) have been reported. In this study, we consider a One Health approach to investigate yaws and describe skin ulcers and corresponding *T. pallidum* serology results among children living in the Tarangire-Manyara ecosystem, an area with increasing wildlife-human interaction in northern Tanzania.

**Methods:**

To investigate human yaws in Tanzania, we conducted a cross-sectional study to screen and interview skin-ulcerated children aged 6 to 15 years, who live in close proximity to two national parks with high numbers of naturally *TPE*-infected monkeys. Serum samples from children with skin ulcers were tested for antibodies against the bacterium using a treponemal (*Treponema pallidum* Particle Agglutination assay) and a non-treponemal (Rapid Plasma Reagin) test.

**Results:**

A total of 186 children aged between 6 and 15 years (boys: 10.7 ± 2.1 (mean ± SD), *N* = 132; girls: 10.9 ± 2.0 (mean ± SD), *N* = 54) were enrolled. Seven children were sampled at health care facilities and 179 at primary schools. 38 children (20.4%) reported active participation in bushmeat hunting and consumption and 26 (13.9%) reported at least one physical contact with a NHP. None of the lesions seen were pathognomonic for yaws. Two children tested positive for treponemal antibodies (1.2%) in the treponemal test, but remained negative in the non-treponemal test.

**Conclusions:**

We found no serological evidence of yaws among children in the Tarangire-Manyara ecosystem. Nevertheless, the close genetic relationship of human and NHPs infecting *TPE* strains should lead to contact prevention with infected NHPs. Further research investigations are warranted to study the causes and possible prevention measures of spontaneous chronic ulcers among children in rural Tanzania and to certify that the country is free from human yaws.

## Background

The bacterium *Treponema pallidum* subsp. *pertenue* (*TPE*) causes a multi-stage disease called yaws, which is commonly found in tropical areas with high rainfall and humidity. Children below the age of 15, living in rural areas with poor hygienic conditions are predominantly affected [1]. In 1948, there were between 50 to 150 million yaws cases globally [1]. African countries such as Ghana, Ivory Coast, Cameroon and the Democratic Republic of the Congo reported more than 100,000 cases annually [1]. The first yaws eradication campaign reduced the prevalence of yaws by 95%. In recent years, however, yaws has reemerged and is currently subject to a second, ongoing eradication campaign. The epidemiological status of Tanzania and 75 other countries with a known history of human yaws is currently unknown [2]. In the 1950s, there were an estimated 61,800 yaws cases in Tanganyika and 5400 cases in Zanzibar [3], the two countries that united to form Tanzania in 1964. Available reports show that the disease was common in Tanzania. High infection rates were for example reported along Lake Tanganyika in the West [3], along Lake Nyasa and Songea in the South [4], and in Tanga, Newala and Lindi in the East [5]. In North Tanzania, few cases of yaws were reported especially among the Maasai and Hadza communities [6]. Records provided by the Tanganyika Director of Medical Services showed that in 1930 and 1950 an estimated 137,000 and 52,400 yaws patients respectively received treatment across the country [3]. Like in many other yaws-endemic countries, there was a dearth of scientific studies to evaluate the epidemiology of yaws. As a result, the majority of reported prevalence data from Tanzania were derived from health facilities and ‘colonial traders’ records. There is a possibility that yaws statistics are biased due to limitations in documentation in most health facilities, limited access to primary health care for all patients, and false-positive reports due to cross-reacting antibodies to syphilis. The last national yaws report to the WHO in 1978 describes only 77 human yaws cases [7]. Recent studies that evaluate the successful elimination of human yaws in Tanzania do not exist. However, skin ulcers among children are still commonly seen in rural areas of Tanzania [8].

Human yaws initially presents with a skin ulcer that occurs at the invasion site. The skin lesion pathognomonic to the primary stage of yaws (mother yaws) is typically painless. Sometimes lesions are itchy with raised dark margins, an erythematous moist center and they are commonly covered with crust [9]. Other lesions typical in the primary stage are small yellow skin bumps and solitary erythematous papules of two to five centimeters in diameter and squamous macules. The primary skin ulcer is highly infectious and it can take weeks or months to heal spontaneously, leaving behind a hypopigmented or pressed down lesion demarcated by a darker border [9]. After the primary stage lesion has healed, the secondary stage ensues with typical lesions including painful digital swellings and palmar and plantar cracks or discolorations [10]. If untreated these lesions will heal spontaneously after weeks or months and the patient likely enters a latent stage, where evidence of infection can only be obtained through serological tests. Relapses of secondary manifestations have been reported after 5 to 10 years (as reviewed in [10]). If untreated, ~ 10% of patients will proceed to the tertiary stage, which can result in subcutaneous gummatous nodules and chronic periostitis [10]. Transmission occurs through direct contact with infectious lesions.

While data on human yaws are currently not available from Tanzania, infection of nonhuman primates (NHPs) with *TPE* have been reported [11]. The disease is widespread in NHPs and involves at least four species, olive baboons (*Papio anubis*), yellow baboons (*Papio cynocephalus*), vervet monkeys (*Chlorocebus pygerythrus*), and blue monkeys (*Cercopithecus mitis*) [12]. Due to the zoonotic potential of *TPE* strains of NHP origin [13] there is the urgent need to apply a One Health approach to countries that report NHP infection but lack current information on human yaws. The One Health approach dictates that human and animal health are interconnected and should be studied in conjunction [14].

We hypothesized that *TPE* infection is present in children living at the NHP-human interface in rural Tanzania and predicted that a proportion of children with skin ulcers have antibodies against *T. pallidum* (*TP*). We present a description of the skin ulcers in Tanzanian children and report the corresponding serological results. Our study was designed to investigate the presence, but not to estimate the prevalence of yaws disease in Tanzanian children with a required accuracy.

## Methods

### Study design, setting and enrollment of participants

**Fig. 1:**
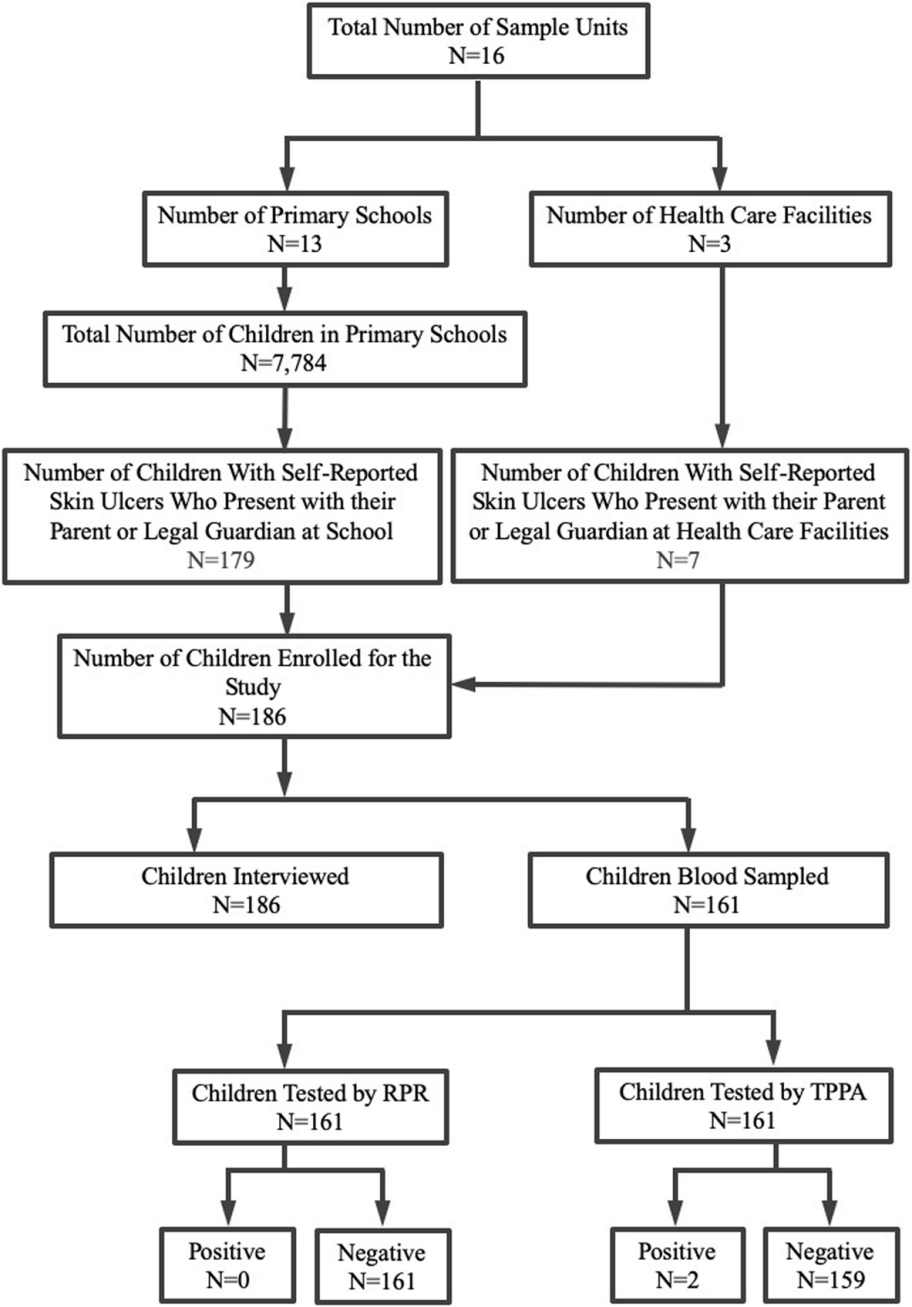
Flowchart illustrating the number of study participants and serological test results

We used a cross-sectional study design to screen for *TP* infection among children who live in close proximity to wildlife areas in Tanzania where NHPs are infected with *TPE* [12, 15, 16]. In this first study we selected two areas in the vicinity to Lake Manyara National Park (LMNP) and Tarangire National Park (TNP), respectively (Additional File 1). Both areas are located in a region that reports increasing wildlife-human conflicts [17]. Since *TPE* infection is generally more common in children of primary school age [18], the study involved children between the age of 6 to 15 years from 13 primary schools and three primary health care facilities (Fig. 1). Enrollment took place from November 2017 to February 2018. Further details on the different institutions are provided in Additional File 2. After a short session of health education on yaws, conducted at the primary schools, we asked children to discuss their skin ulcers at home and to self-report their skin ulcers in the presence of their parents or legal guardians on the following day. Only children whose parents or legal guardians came to school and submitted their written parental consent and who in addition to the parental consent expressed their verbal assent to participate, were enrolled and examined by the study team to confirm the self-reported skin ulcers. Children who were brought to health facilities for outpatient services and who had skin ulcers were asked by the attending clinician in the presence of their parent or legal guardian whether or not they were willing to participate in the study. In case the child and parent/legal guardian agreed, the research team was called to visit the health care facility and the same standards (written consent from parents or a legal guardian and the children’s verbal assent) were applied before children were allowed to enroll into the study. Caution was taken to avoid double enrollment by asking the child and parent or legal guardian about previous participation. Skin ulcers of children at schools and health care facilities were cleaned and dressed. In a primary school setting, we advised the parents or guardians to present their child to a clinician at a nearby health facility to receive further treatment for nonhealing wounds. Figure 1 provides a flowchart summarizing the key features of the study.

### Sample size

From previous studies conducted in Ghana, one of the current yaws endemic countries, the prevalence of yaws among primary school children (with and without skin ulcers) was estimated at 2.5% [19]. In a first approach, we used this value as a design prevalence to calculate the required sample size that was needed to rule out the existence of yaws infection with an error probability of 5% in the subpopulation of children with skin ulcers in Tanzania. We calculated the sample size using the 1-Stage Freedom analysis tool implemented into Epitool software [20, 21], setting the parameters as follows: population size was set to infinity, an error probability of 0.05 was used and the required test sensitivity and specificity of the Serodia *Treponema Pallidum* Particle Agglutination Assay (Serodia TPPA, FujireBio INC, Japan), which was used to screen for the presence of anti-*T. pallidum* antibodies, was set to 100% each, based on data provided by the manufacturer [22]. Under these conditions, a required sample size of at least 119 children resulted. If the design prevalence was strengthened to 2.0%, the minimum sample size amounted to 149 children. In both cases, design prevalence at 2.5 and 2.0%, the actual sample size in the study exceeded the calculated values.

### Study procedures

Children were interviewed by a Tanzanian trained study physician (C.K.C.L.) using a standardized questionnaire that included questions on demographic data (age, sex, education, residence and details on the household), duration and possible causes of their skin ulcer(s), example given an initial injury as well as any history of pain and changes in clinical manifestations. Interviews were conducted in Swahili. A recent study in Central Africa reported frequent physical contact between NHPs and humans [23]. Hence, particular attention was given to information on skin ulcers in other members of the household and the child’s lifetime history of physical contact with NHPs and their body fluids or excrements. We furthermore asked the children whether their family keeps livestock.

After the completion of the interview, up to five ml whole blood was collected under aseptic conditions using a closed blood collection system (Vacutainer, Becton Dickinson Medical, USA). Blood samples were subsequently centrifuged at 2000 xg for 15 min at ambient temperature. Aliquots of 1.8 ml serum were collected in cryovial tubes and were kept in liquid nitrogen until they arrived in the laboratory where they were stored at − 80 °C.

Skin ulcers were photographed and documented as shown in the Yaws Recognition Booklet for Communities published by the World Health Organization [24]. Photographs included an additional length standard and sample ID. While the information obtained from the questionnaires was paired with the respective individuals’ blood sample, any personal details that could be used to identify the child were anonymized. All sampling procedures were performed by a trained and licensed physician.

### Serology

Work steps with potentially infectious material were performed under a BSL-2 laminar flow bench. We used treponemal and nontreponemal tests to test for the presence of anti-*T. pallidum* antibodies.

#### Treponemal test

We ran all samples in duplicates using the Serodia TPPA following the manufacturer’s protocol. The test detects serum antibodies directed against *TP*. We considered the assay valid when the positive control was positive at a dilution of 1:320, the sample diluent reacted negatively with both sensitized and unsensitized particles, and each specimen reacted negatively with unsensitized particles at the final dilution of 1:40. Two researchers interpreted the results independently following the manufacturer’s interpretation guidelines. Samples that reacted with both sensitized and unsensitized particles were reanalyzed following an absorption procedure described in the assay’s manual.

#### Non-treponemal test

Serum samples were tested using the Rapid Plasma Reagin test (RPR, Bio–Rad, France), following the manufacturer’s instructions. Non-treponemal tests (NTTs) detect antibodies directed against lipoidal antigens from both treponemes and damaged host cells [25]. Test results were interpreted according to the manufacturer’s interpretation guidance by two independent researchers. We considered the assay valid, when the positive control (human serum containing antibodies against-*T. pallidum*) showed agglutination and the negative control (rabbit serum in phosphate buffer) of the test kit showed no agglutination. We categorically excluded samples where the no-template control (NTC) did not remain negative.

### Statistical analysis

Statistical analysis was performed utilizing Prism 8 (GraphPad Software Inc). Comparison of categorical variables such as bushmeat hunting and contact to NHPs was done by using 2 × 2 contingency tables and a two-tailed Fisher’s exact test. Proportions were tested at a significance level of 0.05. For prevalence estimation, the upper-limited one-sided 95% confidence intervals (CIs) were calculated using the Wilson/Brown method.

## Results

### Socio-demographic data

A total of 186 children aged between 6 and 15 years (boys: 10.7 ± 2.1 (mean ± SD), *N* = 132; girls: 10.9 ± 2.0 (mean ± SD), *N* = 54; Fig. 1, Additional File 3) were sampled at their respective schools in the Tarangire-Manyara ecosystem. A mean of 11.6 (±8.6 SD) children was sampled at each of the 16 locations (Additional File 2) where the study was conducted. Children attended different classes at primary school and all lived in villages that were close to wildlife protected areas. The median proximity of the sampling location to the nearest border of a national park that harbors *TPE* infected NHPs was 15.6 km (25th–75th percentile 3.8–31.9, *N* = 16 locations; Distance and Area Measure Software version 1.2.4). Of all 186 children with skin ulcers, 38 children (20.4%) reported active participation in bushmeat hunting and consumption, 26 (13.9%) reported to have had at least one physical contact with a NHP, and 84 children (45.1%) lived in households that keep livestock. Among children with skin ulcers, those who participate in bushmeat hunting and consumption were significantly more often exposed to physical contact with a NHP (*N* = 186, odds ratio 4.4 [two-sided 95% CI 1.9–10.2]; *p* = 0.0013; 2-tailed Fisher’s exact test). Thirty children (16.1%) reported that other family members of their household had similar skin lesions.

### Skin ulcers

**Fig. 2:**
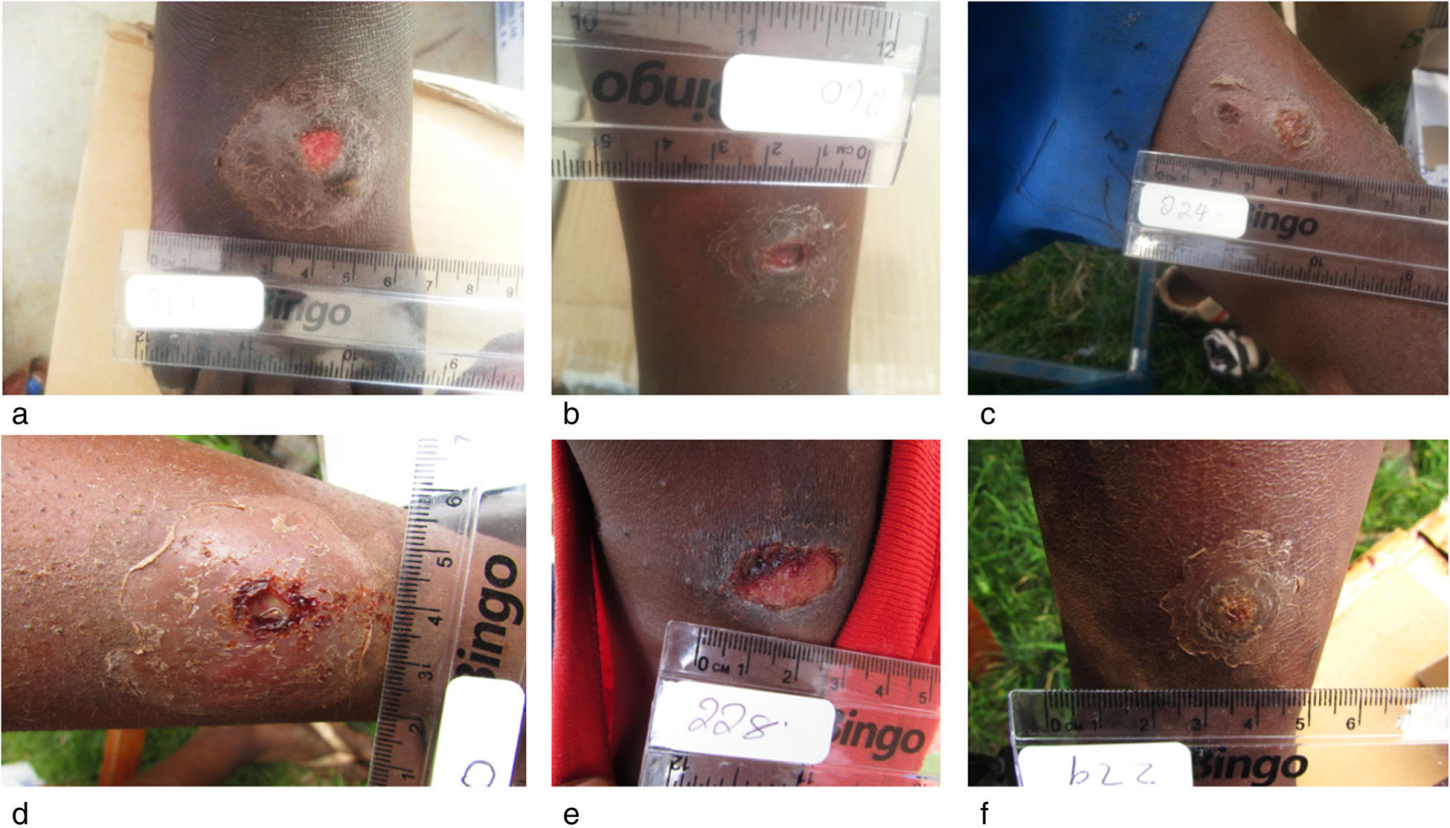
Representative skin ulcers in primary school children from Tarangire-Manyara ecosystem. **a**, **b**, **c**, **d**, and **f** are on lower limbs, (**e**) is on the neck. The reported duration of the skin lesions was (**a**): 60 days, (**b**): 7 days, (**c**): 7 days, (**d**): 14 days, (**e**): 14 days, and (**f**): 14 days. A representative figure for one of the bigger range ulcers (> 5 cm) has been included in the Additional Materials (Additional File 4)

In our subpopulation of skin ulcerated children, we had more boys (*N* = 132, 71%) than girls (*N* = 54, 29%)**.** The ulcers appeared in various shapes, ranging in size between approximately one to five centimeters (Fig. 2, Additional File 4). Pathognomonic yaws lesions were not seen in our group of children. The frequency distribution of the skin ulcer location from this study is illustrated in Additional File 3. Unfortunately, not all data were available from all ulcers. Some children, for example, were unable to answer the question on how long they experienced the skin ulcer or technical caveats in combination with time management prevented the study team from documenting ulcer characteristics. In the following, we consistently report data that were available to us. 24.7% (*N* = 46/186) of the children reported that their ulcers are painless. The median duration of ulcers at examination was 14 days (25th–75th percentile 7–30, *N* = 183 ulcers). Twelve (6.4%) children reported the presence of their skin ulcer for 1 year or more. On the cause of the ulcers, 58.1% (*N* = 108/186) of the children reported that their ulcers originated from injuries acquired during sports or daily activities at home or school. However, 41.9% (*N* = 78/186) reported that their skin lesions started as papules of unknown cause and later developed into skin ulcers. Majority of the ulcers were circular in shape (69.9%, *N* = 79/113), had regular margins (85.0%, *N* = 96/113) and sloping edges (97.3%, *N* = 110/113). Most of them (58.4%, *N* = 66/113) were healing, with health granulating tissue on the floor and serous discharge or no discharge. However, 37.2% (*N* = 42/113) were spreading, covered with slough or scabs, with purulent or serosanguinous discharge. The remaining ulcers (4.4%, *N* = 5/113) were callous, with chronic unhealthy granulation tissue.

### Serology

The *T. pallidum* specific Serodia TPPA test generated two positives out of a total of 161 tested sera [1.2%; one-sided upper 95% confidence limit = 3.86%])*.* The samples came from a seven and a thirteen-year-old boy at Mbugwe Primary School in the school district of Babati. No samples, including the two TPPA positives, were positive in the RPR assay. The latter finding, leads to a one-sided upper 95% confidence limit of 1.84% for the prevalence of yaws [one-sided upper 95% confidence limit = 1.84%].

## Discussion

In Tanzania, human yaws was last heard of in 1978 where 77 cases were reported to the WHO [7]. Since then, human yaws seemed to have disappeared most likely due to the effect of mass-treatment of yaws patients and their contacts during the first yaws eradication campaign in the 1950s to mid-1960s. However, *TPE* strains have been frequently described in NHPs in Tanzania and are genetically highly similar to human yaws causing strains [11]. Given the close genetic similarity of *TPE* strains of human and NHP origin [11], transmission may occur between NHPs and humans with the highest risk at the NHP-human interface in areas that have a high burden of infected primates. We therefore strategically selected two known hotspots of NHP-*TPE* infection in Tanzania, LMNP and TNP, to screen children for possible yaws infection. Overall, school children are the most affected age group in human yaws [18], which guided our study design. Although the presence of anti-*T. pallidum* antibodies does not allow for the discrimination of any of the three diseases caused by the subspecies *pallidum* (syphilis), *pertenue* (yaws), or *endemicum* (bejel) [10] it is still a very useful tool for mass-screening of yaws as outlined in the Morges Strategy [26]. Although our investigations in Tanzania are ongoing, our findings are supportive of the notion that human yaws is currently not a major health concern in the Tarangire-Manyara ecosystem. Additionally, if NHP to human transmission of yaws occurs, these are most likely very rare events. The WHO is targeting to eradicate yaws globally by the year 2030 [27]. So far only India has been certified free from yaws [28]. In Tanzania, yaws was historically reported from almost the whole country; in the north among Maasai, Hadzabe and nearby communities, in the south in Songea, in the west along Lake Tanganyika, and in the east in Tanga, Newala and Lindi. This study was conducted in the north among Maasai and non-Maasai rural communities. We found skin ulcers to be a common occurrence but there was no serological evidence of yaws. Research is warranted in the remaining areas to identify if any hotspots of infection remain, and if possible certify the county free of yaws.

Only 1.2% of children had antibodies directed against *T. pallidum*. This is similar to findings by Klouman et al., in rural Kilimanjaro, northern Tanzania where the seroprevalence of antibodies against *T. pallidum* in school-aged children was 6.4% among girls and 1.1% among boys [29]. Klouman et al. argue that the most common route of transmission among girls is most likely sexual abuse due to higher prevalence of *TP* antibodies among girls compared to boys, and an additional lack of association between parents’ and children’s sero status. In our study, children who had *TP* antibodies were both boys, 7 and 13 years old. They did not have non-treponemal antibodies when tested by RPR, suggesting that most likely they have been in contact with syphilis and were treated with antibiotics. Less likely, but still debatable is the possibility that the *TP*-seropositive children had a history of yaws and their skin ulcers are of different aetiology.

While our serological results indicate that children in the Tarangire-Manyara ecosystem are most likely not infected with *TPE*, future research should include broad range tests, such as metagenomics, to diagnose the causes of the frequent skin ulcers in children. These efforts should also include the collection of paired serum and lesions swab samples to increase diagnostic sensitivity and specificity. With an error probability of 0.05, we can conclude that if human yaws is present in children with skin ulcers in the Tarangire-Manyara ecosystem, its true prevalence was below 3.86% (based on TPPA results) and 1.84% (based on the results of the RPR assay), respectively. Moreover, since the study population is given by children with skin ulcers, which are an important indicator for active yaws infection, the actual prevalence of yaws in the whole population of children, might be even lower in numbers.

The impression that the majority of the skin ulcers are a result of tissue trauma that became infected is also supported by the finding that most ulcers were located at the lower extremities (Additional File 3) and showed the typical morphology of the skin lesions. Most children only had a single chronic skin lesion which was atypical for yaws and not comparable to lesions reported from yaws endemic areas such as Vanuatu [24]. The majority of the ulcers were teeming with pus, painful, foul smelling, swollen or had delayed healing. It became obvious that most of the children were at risk of skin infections such as pyodermas, fungal infections and scabies due to poor hygienic conditions [8]. Skin lesions were neither protected from the environment nor regularly cleaned. In sum, this added to morbidity on children as painful ulcers restricted their participation in daily activities at school and at home. Additionally, unchecked infection could lead to debilitating conditions such as osteomyelitis that carries a risk of permanent disability. Some of the documented ulcers in this study resemble morphological characteristics seen in skin ulcers caused by *Haemophilus ducreyi*. The bacterium is regionally known to mimic yaws-infection in children and has been identified as one of the major differential diagnosis for human yaws infection [30]. Future studies that aim to investigate the causes of skin lesions in children in Tanzania should therefore specifically test for the presence of *H. ducreyi*. Yet, treponemes are a genus of bacteria that infects humans and animals. Many species of the genus are currently described exclusively on the 16S RNA gene level and it must be assumed that a substantial number of *Treponema* species is yet undiscovered. Children in Tanzania are frequently exposed to livestock and their excrements (skin ulcer contamination risk). Since livestock is known to harbor a number of *Treponema* species that colonize the gastro-intestinal-tract or cause hoof diseases [31], we include the information of livestock contact into our survey. We considered the possibility that closely, not yet discovered *Treponema* spp. could challenge the interpretation of test results. This, however, was not an issue in the current study.

There are several limitations associated with the present study, some of which have already been addressed. Based on the variable prevalence rates documented for latent yaws in endemic yaws communities, more studies with an unbiased study design should be encouraged to equally include children with and without skin ulcers as well as their contacts. Sample size is often a limiting factor associated with restricted resources. Moreover, we acknowledge that the Serodia TPPA specificity and sensitivity values that were used to calculate the sample size can be discussed critically since the outcome of interest in this study is based on the treponemal (Serodia TPPA) and non-treponemal test (RPR). Last, future studies would benefit from validation of the standardized questionnaire that is used for interviews.

## Conclusions

The presence of chronic spontaneous ulcers among children in Tanzania points towards an unnoticed or a neglected health concern in rural communities, which is sustained by low standards of living. We found no serological evidence of yaws infection in children in Tarangire-Manyara ecosystem. Further studies that elucidate the cause and preventive measures for these ulcers are of public health importance. Furthermore, for a sustainable eradication of human yaws, it will be essential to understand whether NHP infecting *TPE* strains are epidemiologically connected to human infection. Feasible interspecies transmission routes between humans and NHPs exist, e.g., through direct contact during bushmeat butchering [23] or indirect contact through vectors such as necrophagous flies [32]. However, the proof of the existence or nonexistence of transmission events between humans and NHPs requires more data from other ecosystems endemic for NHP *TPE* infection. Our results demonstrate a significant relationship between history of hunting and increased likelihood of coming in physical contact with NHPs (*p* = 0.0013). Although we have found no evidence for *TPE* infection in the children in this study, the close genetic relationship of human and NHP infecting *TPE* strains should lead to contact prevention with infected NHPs e.g., during bushmeat hunting as it puts people at risk to theoretically acquire *TPE* infection. Funders are encouraged to specifically support epidemiological studies that are needed to investigate disease prevalence and which help governments and health care institutions to gain data that allow to certify countries free of yaws. At the same time and based on the recent yaws cases in the Philippines, which are the first reported cases in the Philippines since the 1970s [33], the importance of a robust yaws surveillance cannot be stressed enough.

## Supplementary information

**Additional File 1.** Map showing Lake Manyara and Tarangire National Park (yellow areas) and the 13 primary schools (red dots) and an additional three primary health care facilities (red cross) where children were samples. The corresponding GPS data can be found in the Additional File 2. The map was constructed using QGIS 3.10.2-A Coruña with open access map source Bing Aerial©Microsoft (http://ecn.t3.tiles.virtualearth.net/tiles/a {q}.jpeg?g = 1).

**Additional File 2.** Sampling areas and the number of children enrolled from each sampling location.

**Additional File 3. (A)** Population pyramidic graph of the age distribution in children that were enrolled in this study. The total number of girls (green bars) was 54 compared to 132 boys (blue bars). **(B)** Distribution and frequency of 196 skin ulcer locations in 186 children sampled in this study. Picture source human silhouette (modified, Pixabay [Internet]. Available from: https://pixabay.com/de/illustrations/junge-menschliche-silhouette-kinder-2676579/).

**Additional File 4. **Representative skin ulcers greater than 3 cm. (**A**) lower limb, 14-year-old girl (**B**) lower limb, 13-year-old boy (**C**) lower limb, 14-year-old girl (**D**) neck, 14-year-old girl. The reported duration of the skin lesions was (**A**): 366 days, (**B**): 14 days, (**C**): 30 days and (**D**): unknown.

## Data Availability

The raw data are available upon reasonable request from the corresponding author.

## Abbreviations

LMNP: Lake Manyara National Park
NHPs: Nonhuman primates
NTT: Non-treponemal test
RPR: Rapid Plasma Reagin
SUA: Sokoine University of Agriculture
TNP: Tarangire National Park
*TP*: *Treponema pallidum*
*TPE*: *Treponema pallidum* subsp. *pertenue*
TPPA: *Treponema pallidum* Particle Agglutination
WHO: World Health Organization
