# Supplementary material for: Serosurvey of Treponema pallidum infection among children with skin ulcers in the Tarangire-Manyara ecosystem, northern Tanzania
Source: BMC Infect Dis. 2020 Jun 3;20:392. doi: 10.1186/s12879-020-05105-4 (PMC7268494; doi:10.1186/s12879-020-05105-4)
Supplement: Supplementary file 1 — Additional File 1. Map showing Lake Manyara and Tarangire National Park (yellow areas) and the 13 primary schools (red dots) and an additional three primary health care facilities (red cross) where children were samples. The corresponding GPS data can be found in the Additional File 2. The map was constructed using QGIS 3.10.2-A Coruña with open access map source Bing Aerial©Microsoft (http://ecn.t3.tiles.virtualearth.net/tiles/a {q}.jpeg?g = 1). [file 12879_2020_5105_MOESM1_ESM.pdf]

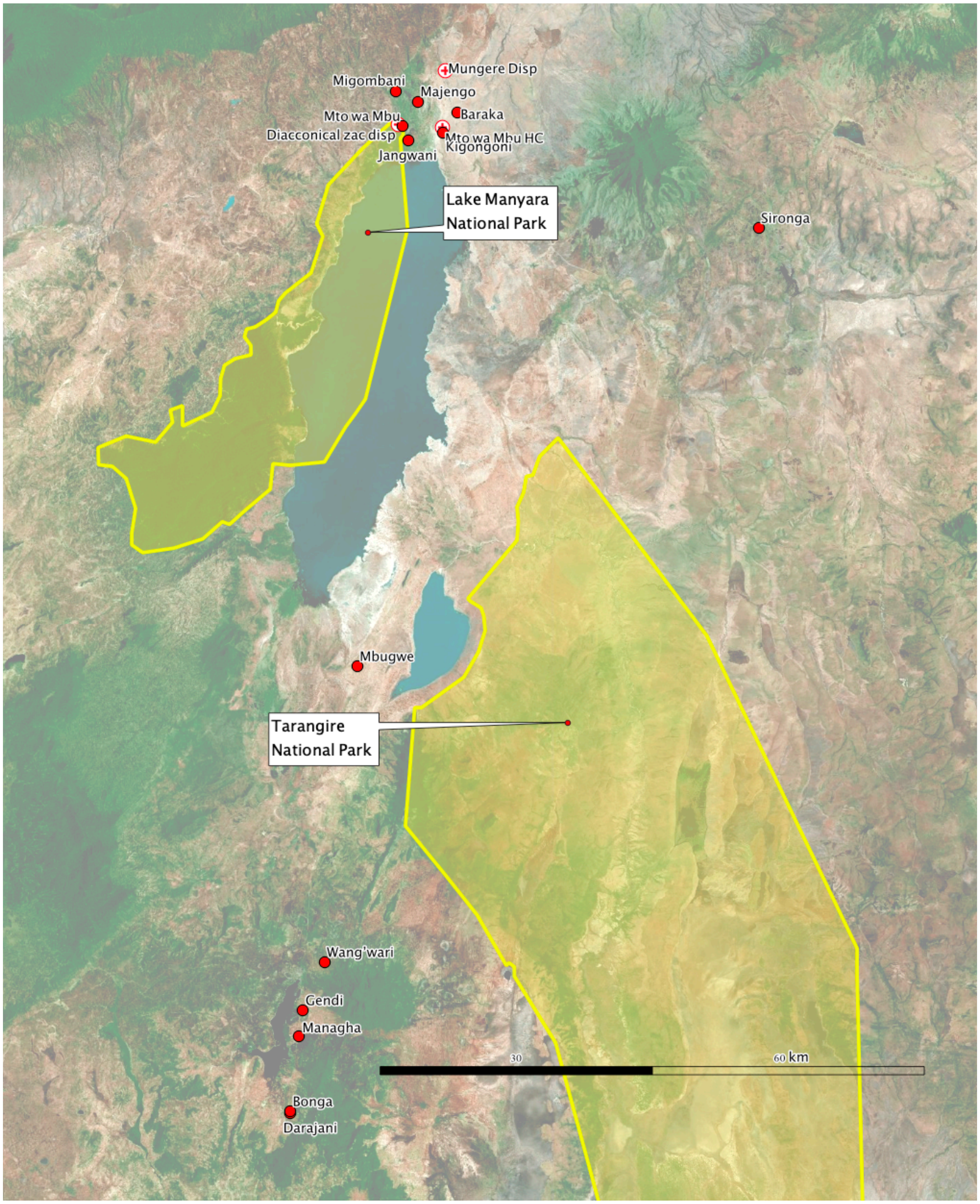

Additional File 1: Map showing Lake Manyara and Tarangire National Park (yellow areas) and the 13 primary schools (red dots) and an additional three primary health care facilities (red cross) where children were samples. The corresponding GPS data can be found in the Additional File 2. The map was constructed using QGIS 3.10.2-A Coruña with open access map source Bing Aerial ©Microsoft (http://ecn.t3.tiles.virtualearth.net/tiles/a{q}.jpeg?g=1).
