# Supplementary material for: Serosurvey of Treponema pallidum infection among children with skin ulcers in the Tarangire-Manyara ecosystem, northern Tanzania
Source: BMC Infect Dis. 2020 Jun 3;20:392. doi: 10.1186/s12879-020-05105-4 (PMC7268494; doi:10.1186/s12879-020-05105-4)
Supplement: Supplementary file 2 — Additional File 2. Sampling areas and the number of children enrolled from each sampling location. [file 12879_2020_5105_MOESM2_ESM.pdf]

Additional File 2. Sampling areas and the number of children enrolled at each sampling location. Schools were not selected on the basis of their exact distance to the respective wildlife area. Instead, we used schools that were accessible to us and that were close to Lake Manyara National Park or Tarangire National Park. The proximity of the facility is only a proxy for the pupils' place of residence.

	**District**	**Location Name**	**GPS Coordinates**		**Total Number of Children**	**Number of Children Enrolled**
**Primary schools**	Monduli	Baraka	-3.359530	35.904170	620	7
		Majengo	-3.348670	35.865460	708	14
		Migombani	-3.338850	35.844140	224	24
		Mto wa Mbu	-3.372998	35.850650	1,117	7
		Sironga	-3.473690	36.202960	142	12
		Kigongoni	-3.379840	35.890580	951	14
		Jangwani	-3.387460	35.855600	533	12
	Babati	Mbugwe	-3.906720	35.805310	701	27
		Darajani	-4.348190	35.738810	543	14
		Wang'wari	-4.198860	35.772870	462	6
		Bonga	-4.346190	35.738990	821	9
		Gendi	-4.246420	35.751670	613	4
		Managha	-4.272220	35.747770	349	29
**Total**	**2**	**13**			**7,784**	**179**
**Health centre (HC) Dispensary (Disp)**	Monduli	Diacconical zac disp	-3.371090	35.845940	unknown	2
		Mungere Disp	-3.317800	35.893000	unknown	2
		Mto wa Mbu HC	-3.374327	35.889823	unknown	3
**Total**	**1**	**3**	**-**	**-**	unknown	**7**
**Grand total**	**2**	**16**	**-**	**-**	**-**	**186**
