# Supplementary material for: Serosurvey of Treponema pallidum infection among children with skin ulcers in the Tarangire-Manyara ecosystem, northern Tanzania
Source: BMC Infect Dis. 2020 Jun 3;20:392. doi: 10.1186/s12879-020-05105-4 (PMC7268494; doi:10.1186/s12879-020-05105-4)
Supplement: Supplementary file 3 — Additional File 3. (A) Population pyramidic graph of the age distribution in children that were enrolled in this study. The total number of girls (green bars) was 54 compared to 132 boys (blue bars). (B) Distribution and frequency of 196 skin ulcer locations in 186 children sampled in this study. Picture source human silhouette (modified, Pixabay [Internet]. Available from: https://pixabay.com/de/illustrations/junge-menschliche-silhouette-kinder-2676579/). [file 12879_2020_5105_MOESM3_ESM.pdf]

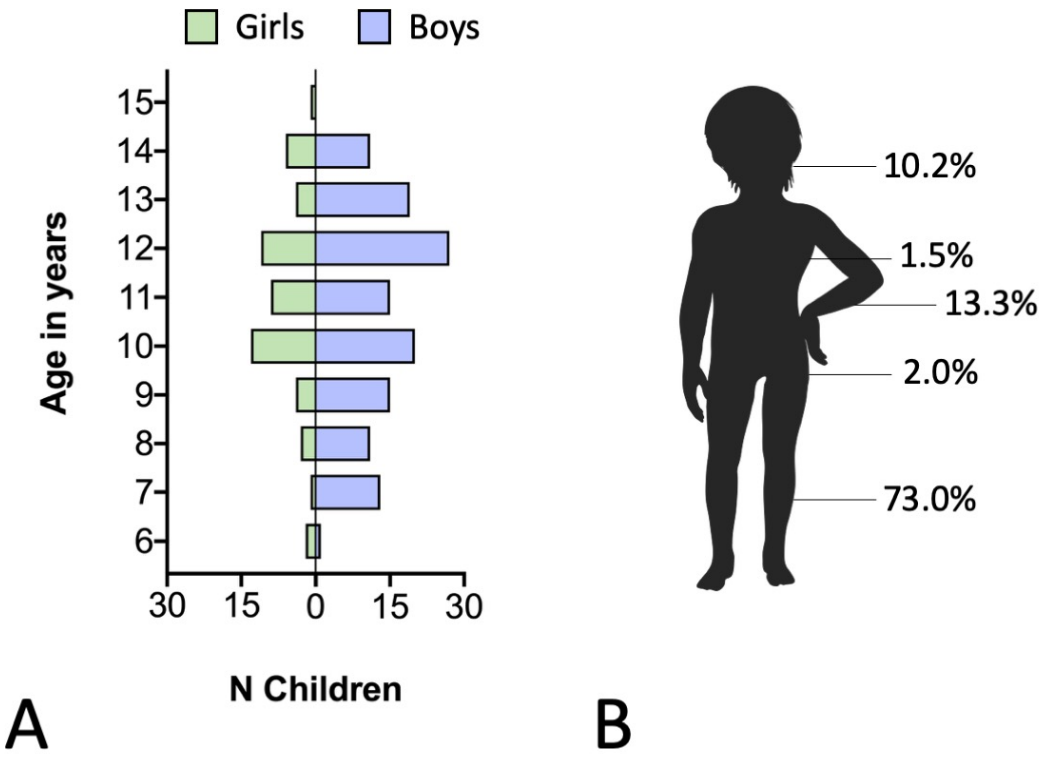

Additional File 3. (A) Population pyramidic graph of the age distribution in children that were enrolled in this study. The total number of girls (green bars) was 54 compared to 132 boys (blue bars). **(B)** Distribution and frequency of 196 skin ulcer locations in 186 children sampled in this study. Picture source human silhouette (modified, Pixabay, available from: https://pixabay.com/de/illustrations/junge-menschliche-silhouette-kinder-2676579/).
