# Supplementary material for: Serosurvey of Treponema pallidum infection among children with skin ulcers in the Tarangire-Manyara ecosystem, northern Tanzania
Source: BMC Infect Dis. 2020 Jun 3;20:392. doi: 10.1186/s12879-020-05105-4 (PMC7268494; doi:10.1186/s12879-020-05105-4)
Supplement: Supplementary file 4 — Additional File 4. Representative skin ulcers greater than 3 cm. (A) lower limb, 14-year-old girl (B) lower limb, 13-year-old boy (C) lower limb, 14-year-old girl (D) neck, 14-year-old girl. The reported duration of the skin lesions was (A): 366 days, (B): 14 days, (C): 30 days and (D): unknown. [file 12879_2020_5105_MOESM4_ESM.pdf]

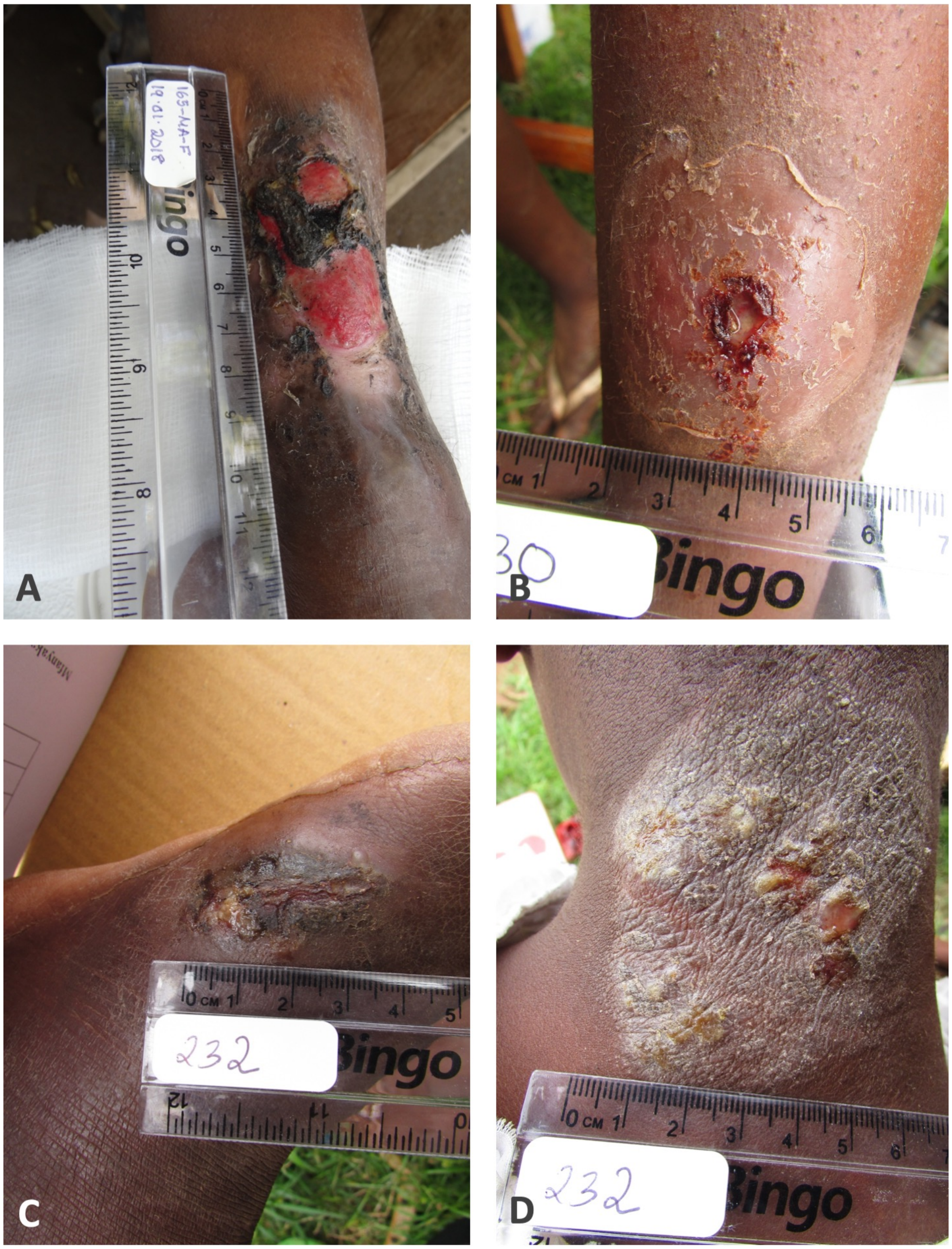

Additional File 4: Representative skin ulcers greater than 3 cm. (A) lower limb, 14-year-old girl **(B)** lower limb, 13-year-old boy **(C)** lower limb, 14-year-old girl **(D)** neck, 14-year-old girl. The reported duration of the skin lesions was (**A**): 366 days, (**B**): 14 days, (**C**): 30 days and (**D**): unknown.
